# A dataset of labelled objects on raw video sequences

**DOI:** 10.1016/j.dib.2020.106701

**Published:** 2020-12-26

**Authors:** Hyomin Choi, Elahe Hosseini, Saeed Ranjbar Alvar, Robert A. Cohen, Ivan V. Bajić

**Affiliations:** a School of Engineering Science, Simon Fraser University, Burnaby, BC V5A 1S6, Canada

**Keywords:** Object detection, Video coding, Video compression, Video coding for machines

## Abstract

We present an object labelled dataset called SFU-HW-Objects-v1, which contains object labels for a set of raw video sequences. The dataset can be useful for the cases where both object detection accuracy and video coding efficiency need to be evaluated on the same dataset. Object ground-truths for 18 of the High Efficiency Video Coding (HEVC) v1 Common Test Conditions (CTC) sequences have been labelled. The object categories used for the labeling are based on the Common Objects in Context (COCO) labels. A total of 21 object classes are found in test sequences, out of the 80 original COCO label classes. Brief descriptions of the labeling process and the structure of the dataset are presented.

## Specifications Table

**Table:** 

Subject	Computer Vision and Pattern Recognition
Specific subject area	Object detection, object classification, video compression
Type of data	Annotations
How data were acquired	Data was created by processing and analyzing HEVC v1 CTC test video sequences. The sequences were first passed through YOLOv3 object detector to find initial object locations and classes. Then this information was refined manually, frame by frame, using the Yolo_mark software tool, to create the final object labels.
Data format	Analyzed
Parameters for data collection	Raw primary data was converted from YUV420 to RGB24 format prior to data collection.
Description of data collection	The converted RGB24 data were passed through YOLOv3 object detector to find initial object locations and classes. Then this information was refined manually, frame by frame, using the Yolo_mark software tool, to create the final object labels.
Data source location	Institution: Simon Fraser University
	City/Town/Region: Burnaby, British Columbia
	Country: Canada
	Latitude and longitude (and GPS coordinates, if possible) for collected samples/data: Latitude: 49.276765, Longitude: −122.917957
	Primary data sources:
	Raw HEVC v1 CTC video sequences maintained by ITU-T JCT-VC:
	https://www.itu.int/en/ITU-T/studygroups/2017-2020/16/Pages/video/jctvc.aspx)
Data accessibility	Repository name: Mendeley
	Direct URL to data: http://dx.doi.org/10.17632/hwm673bv4m.1
	Instructions for accessing these data:
	Secondary data, which this paper describes, is publicly available at the above URL.
	https://www.itu.int/en/ITU-T/studygroups/2017-2020/16/Pages/video/jctvc.aspx

## Value of the Data

• We provide a dataset of object labels for raw (uncompressed) HEVC v1 CTC video sequences.
• Our dataset can benefit the research at the intersection of video coding and computer vision. It is one of the datasets used in the MPEG-VCM (Video Coding for Machines) standardization group.
• The dataset can be used to study the impact of video compression on object detection, or for developing and analyzing systems that perform video compression and object detection simultaneously, as in MPEG-VCM.

## 1 Data Description

We present a dataset called **SFU-HW-Objects-v1**, which contains bounding boxes and object class labels for High Efficiency Video Coding (HEVC) v1 Common Test Conditions (CTC) video sequences [1], [2]. The presented dataset contains only object labels; video sequences themselves can be obtained from the Joint Collaborative Team on Video Coding (JCT-VC).^1^ Table 1 lists 18 video sequences in this group, along with their characteristics and the number of objects found in each sequence. Table 2 shows the list of object classes found in these sequences. Object class IDs follow the Common Objects in Context (COCO) [3] label indices.

**Table 1:** HEVC v1 CTC sequences and the number of object classes found in each sequence.

**Class**	**Sequence name**	**Width × Height**	**Frame count**	**Frame rate (Hz)**	**Bit depth**	**Number of object classes**
A	Traffic	2560 × 1600	150	30	8	**2**
A	PeopleOnStreet	2560 × 1600	150	30	8	**4**
B	BQTerrace	1920 × 1080	600	60	8	**9**
B	BasketballDrive	1920 × 1080	500	50	8	**4**
B	Cactus	1920 × 1080	500	50	8	**1**
B	Kimono	1920 × 1080	240	24	8	**2**
B	ParkScene	1920 × 1080	240	24	8	**4**
C	BQMall	832 × 480	600	60	8	**3**
C	BasketballDrill	832 × 480	500	50	8	**4**
C	PartyScene	832 × 480	500	50	8	**6**
C	RaceHorses	832 × 480	300	30	8	**2**
D	BQSquare	416 × 240	600	60	8	**7**
D	BasketballPass	416 × 240	500	50	8	**4**
D	BlowingBubbles	416 × 240	500	50	8	**3**
D	RaceHorses	416 × 240	300	30	8	**2**
E	KristenAndSara	1280 × 720	600	60	8	**3**
E	Johnny	1280 × 720	600	60	8	**3**
E	FourPeople	1280 × 720	600	30	8	**4**

**Table 2:** Object classes found in the HEVC v1 CTC sequences.

**Class ID**	**Object**	**Class ID**	**Object**	**Class ID**	**Object**
0	Person	17	Horse	56	Chair
1	Bicycle	24	Backpack	58	Potted plant
2	Car	25	Umbrella	60	Dining table
5	Bus	26	Handbag	63	Laptop
7	Truck	27	Tie	67	Cell phone
8	Boat	32	Sports ball	74	Clock
13	Bench	41	Cup	77	Teddy bear

**Fig. 1:**
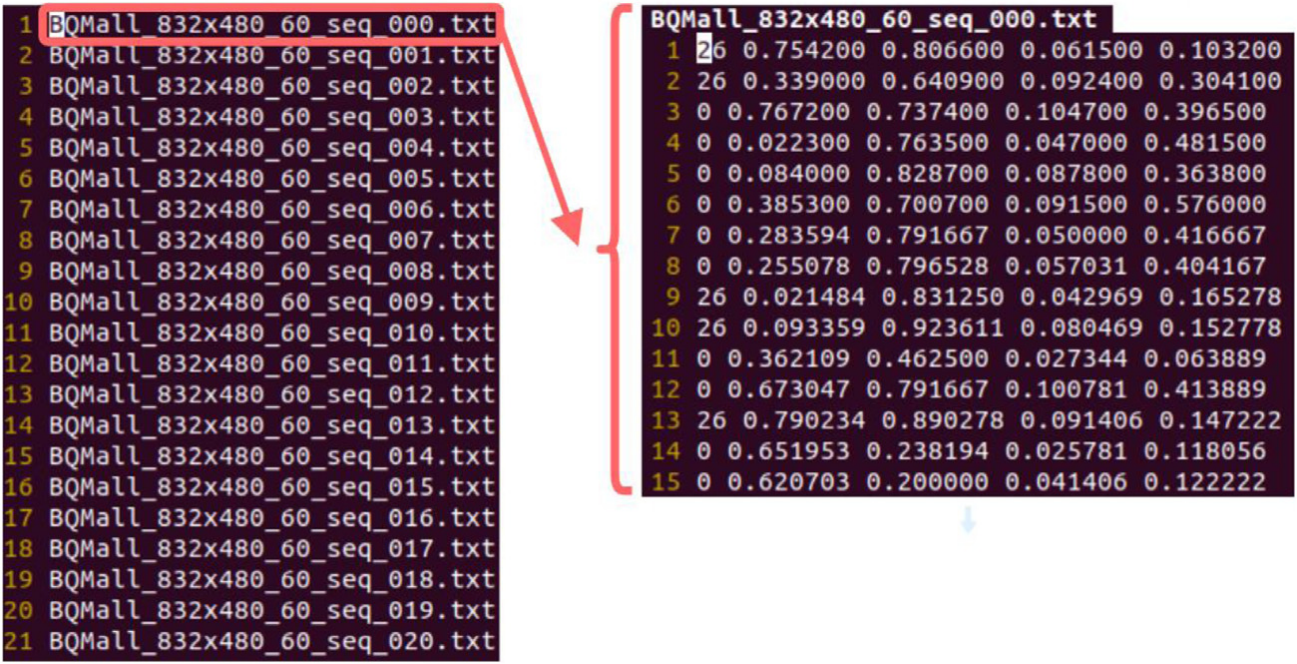
Data files and their contents.

Object labels are organized into separate folders, one for each sequence. Data file structure is shown in Fig. 1. There is one text file per each frame of each sequences containing object labels. The filename indicates the name of the sequence, resolution, frame rate, and the frame index, as shown in Fig. 1. Within the file, each object is annotated by a row in the file. The first element of each row is the object class ID based on the COCO object categories. For example, the first row in the right part of Fig. 1 shows class ID 26, which corresponds to “Handbag” in Table 2.

**Fig. 2:**
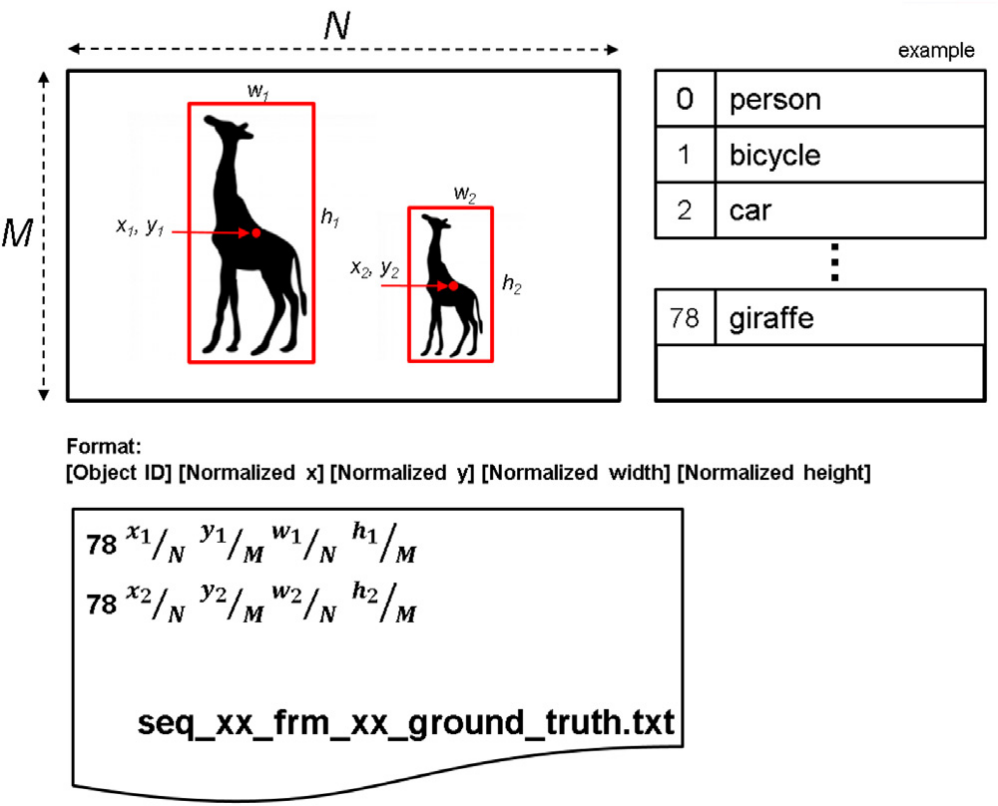
Illustration of the object annotation format.

The remaining four numbers in the row specify the bounding box of the corresponding object. Of these four numbers, the first two specify the center coordinates of the corresponding bounding box, relative to the top-left corner of the video frame, normalized by the resolution, and the last two elements are the width and height of the bounding box, again normalized by the resolution. An example showing how these are computed in presented in Fig. 2. The example shows a frame with two giraffes, so there will be two rows in the annotation file, each starting with index 78 (the COCO class ID for “giraffe”). The big giraffe has a bounding box of width $w_{1}$, height $h_{1}$, centered at $\left(x_{1},y_{1}\right)$ relative to the top-left corner of the frame. Hence, the next for numbers in the annotation record for this giraffe will be

$$x_{1}\;/\;N\;y_{1}\;/\;M\;w_{1}\;/\;N\;h_{1}\;/\;M$$

where M and N are the height and width of the frame, respectively. The small giraffe has a bounding box of width $w_{2}$, height $h_{2}$, centered at $\left(x_{2},y_{2}\right)$ relative to the top-left corner of the frame, so the annotation record for this giraffe will be

$$x_{2}\;/\;N\;y_{2}\;/\;M\;w_{2}\;/\;N\;h_{2}\;/\;M$$

**Fig. 3:**
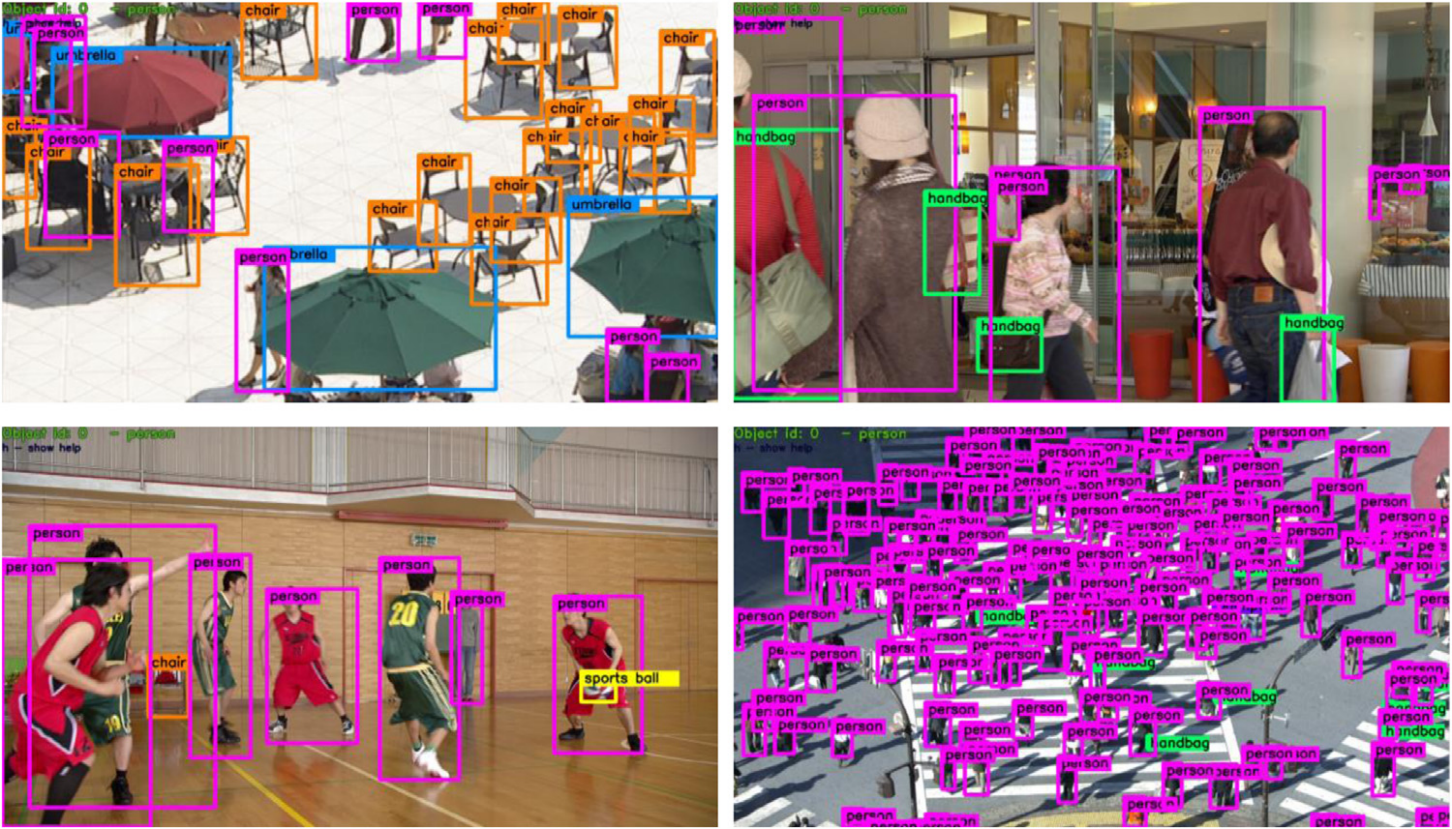
Examples of object annotations overlaid on the corresponding frame.

Fig. 3 shows examples of object annotations overlaid on the corresponding frames. As seen in these examples, many of the sequences in the dataset contain people, but they also contain a variety of other objects. The scale and density of the objects varies significantly among the sequences.

## 2 Experimental Design, Materials and Methods

**Fig. 4:**
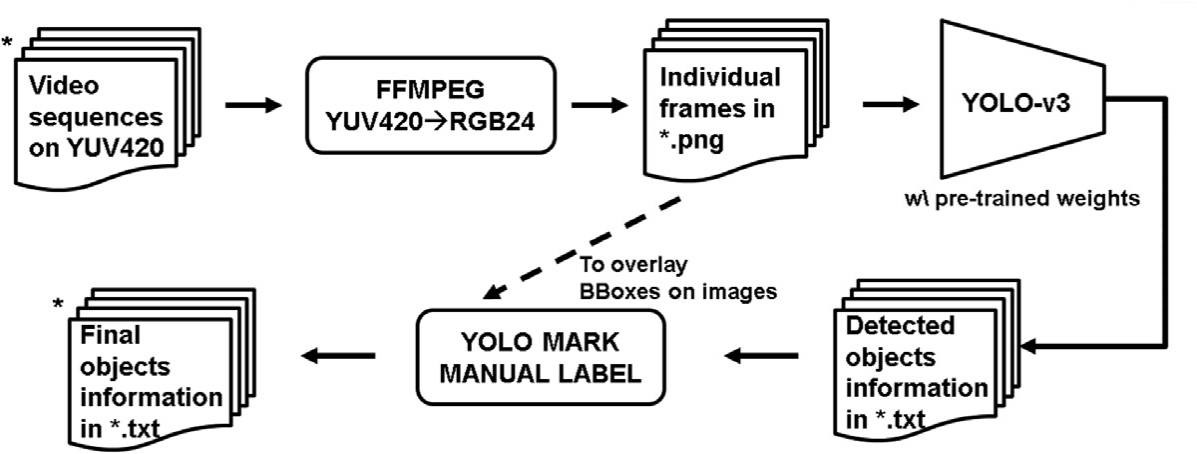
Labeling process.

Raw HEVC v1 CTC video sequences in the YUV420 format are maintained by the Joint Collaborative Team on Video Coding (JCT-VC).^2^ They can be obtained via FTP^3^ following the procedure described in Section 2 of [4]. To create object annotations, we employed a semiautomatic labeling process illustrated in Fig. 4. First, raw YUV420 video sequences were converted to RGB444 (RGB24) and each frame was saved in a separate PNG file. The frames were then fed to the YOLOv3 object detector [5], using the Darknet software from [6], with pre-trained weights^4^ trained on the COCO 2014 dataset, to get the initial object annotations. Then, using the annotation editing tool Yolo_mark,^5^ incorrect positions of the boxes were manually corrected and falsely detected objects were removed. If an object was detected in an earlier frame, and is still visible in the current frame but was not automatically detected by the YOLOv3 object detector, then we manually label it in the current frame and continue labeling it in subsequent frames until the object moves out of view. We also tried to fill in the gaps in detection – if an object was detected in frame n and frame n+k but not in the intermediate k−1 frames, then a box and the corresponding label for this object was added in the intermediate frames.

The final labels and bounding box coordinates were saved to a file corresponding to the current frame. As mentioned before, the annotation file is a text file whose name contains the sequence name, resolution, frame rate, and frame index.

## Ethics Statement

The work did not involve any human or animal subjects, nor data from social media platforms.

## CRediT Author Statement

**Hyomin Choi:** Conceptualization, Methodology, Software, Data curation, Writing - original draft. **Elahe Hosseini:** Methodology, Data curation, Writing - original draft. **Saeed Ranjbar Alvar:** Conceptualization, Investigation, Validation. **Robert A. Cohen:** Supervision, Writing - review & editing. **Ivan V. Bajić:** Supervision, Writing - review & editing, Project administration, Funding acquisition.

## Declaration of Competing Interest

The authors declare that they have no known competing financial interests or personal relationships which have or could be perceived to have influenced the work reported in this article.

## Footnotes

1 https://www.itu.int/en/ITU-T/studygroups/2017-2020/16/Pages/video/jctvc.aspx

2 https://www.itu.int/en/ITU-T/studygroups/2017-2020/16/Pages/video/jctvc.aspx

3 ftp://hevc@mpeg.tnt.uni-hannover.de/testsequences/

4 https://pjreddie.com/media/files/yolov3.weights

5 YOLO Mark, “https://github.com/AlexeyAB/Yolo_mark
